# Targeting pathological brain activity-related to neuroinflammation through scRNA-seq for new personalized therapies in Parkinson’s disease

**DOI:** 10.1038/s41392-024-02086-7

**Published:** 2025-01-10

**Authors:** Daniela Mirzac, Manuel Bange, Sebastian Kunz, Phil L. de Jager, Sergiu Groppa, Gabriel Gonzalez-Escamilla

**Affiliations:** 1 Department of Neurology, Universitätsmedizin Mainz and Saarland University Hospital Homburg, Homburg, Germany; 2 Institute of Immunology, Universitätsmedizin Mainz, Mainz, Germany; 3 Center for Translational & Computational Neuroimmunology, Department of Neurology and the Taub Institute for Research on Alzheimer’s Disease and the Aging Brain, Columbia University Irving Medical Center, New York, USA

**Keywords:** Neurological disorders, Molecular neuroscience

Dear Editor,

Parkinson’s disease (PD), is the most common neurodegenerative movement disorder with robustly identifiable electrophathophysiological hallmarks, namely increased beta and reduced narrow gamma oscillatory activity.^1^ In PD abnormal synchronization of the basal ganglia-thalamo-cortical circuits, which converge on the primary motor and the premotor cortex, is widely accepted to underlie debilitating motor symptoms. Deep brain stimulation (DBS) effectively improves motor symptoms in PD by decreasing cortical beta and increasing gamma activity, as well as modifying gamma-to-beta coupling.^1^ Yet, little is known about the link between pathological oscillatory activity and the underlying molecular signatures of the degenerating brain tissue in PD.

We aimed at investigating the cell-specific gene expression responses to pathological beta and gamma oscillatory activity, including their cross-frequency interactions. For this, we analyzed a total of 101,691 individual cells from a unique ex-vivo dataset, consisting of single-cell RNA sequencing (scRNA-seq) from dorsolateral prefrontal cortex samples from patients that underwent DBS surgery (*n* = 14), from two groups: nine PD and five non-PD patients (see Supplementary material). Cells were grouped into 10 distinct cell types using UMAP (Fig. 1a). In PD, in order of frequency, we observed microglia 47%, oligodendrocytes 39%, OPCs (oligodendrocytes precursor cells) 8%, and astrocytes 2%. To limit the impact of cell heterogeneity we designed well-matched biological replicates avoiding technical noise background, eliminating any significant difference in cell frequency of each cell type between groups (see Supplementary Methods).

**Fig. 1:**
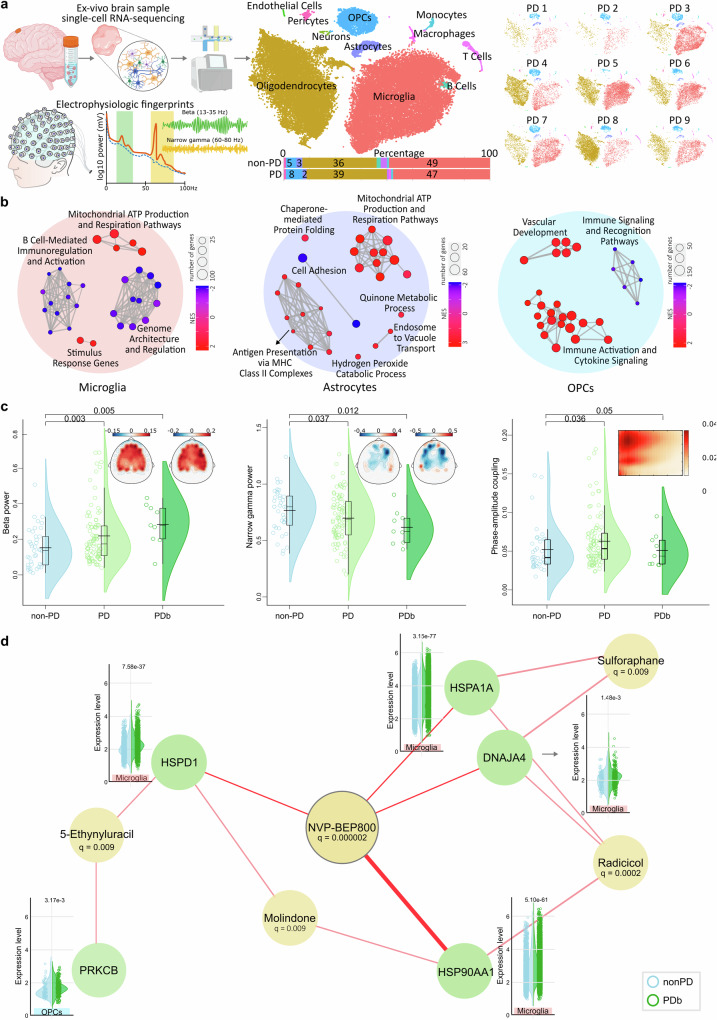
Single-cell underpinnings of electropathophysiological activity and possible therapeutic targets**. a** Depiction of the experimental approach. Left-to-right: surgical retrieval of fresh brain cortical samples for tissue processing, single-cell isolation, single-cell RNA sequencing, and preoperative EEG recordings. After clustering of the integrated single-cell RNA sequencing data, cell types were annotated according to the expression of known marker genes and visualized using t-stochastic neighborhood embedding (tSNE) map right; bar plot distribution by percentage of the identified cell types between cohorts. Here, the recoding of electroencephalographic data used to extract beta and narrow gamma-band oscillations as PD electropathophysiologic hallmarks is also depicted. Partly created with BioRender.com. **b** Differential gene expression analysis between PDb and non-PD (*n* = 5) depicted as enrichment network maps per cell type in microglia (left), astrocytes (middle), and oligodendrocytes precursor cells (OPCs; right), highlighting metabolic and inflammatory pathways abnormally regulated in PD. The size of the circle corresponds to the count of genes in each term, color-coded by normalized enrichment score (NES), and showing only statistically significant results (*p* FDR-adjusted < 0.05). **c** Compared to non-PD (*n* = 38; light blue), PDb (*n* = 9; dark green) showed robustly mirrored the same increased beta power (0.28 ± 0.12, *T* = 2.68, *p* = 0.005) and reduced narrow gamma power (0.62 ± 0.15; *T* = 2.34, *p* = 0.012) as found in larger PD samples (*n* = 91; light green; Beta PD [mean ± sd] 0.22 ± 0.15 vs non-PD 0.15 ± 0.11; *T* = 2.77, *p* = 0.003; narrow gamma PD 0.7 ± 0.21 vs non-PD 0.77 ± 0.19; *T* = 1.8, *p* = 0.037). Group differences in phase-amplitude coupling showed an increase in PD (0.06 ± 0.03; *T* = 1.81, *p* = 0.036) in comparison to non-PD (0.05 ± 0.03), and marginal significance in PDb (0.05 ± 0.02; *T* = 1, *p* = 0.05). **d** Gene-drug interaction analysis using enrichR showed strong drug-gene interactions between 5 genes, with the greatest group differences (PDb vs non-PD), and NVP-BEP800 (*q* = 0.000002), an ATP-competitive inhibitor of Heat-shock protein 90 (HSP90), which could be used for the development of new treatment strategies for PD. Bonferroni correction within each cell type was used to correct the *p*-value. EEG electroencephalography, OPCs oligodendrocytes precursor cells, PD Parkinson’s disease, NES normalized enrichment score, PDb PD subjects with brain biopsies

Group differences in the gene expression attested using gene set enrichment analysis (GSEA) according to Gene Ontology (GO) terms (see “Methods” section) showed dysregulated pathways in four main cell types as a transcriptomic fingerprint for PD (Fig. 1b). In microglia increased activity in mitochondria and protein folding processes appeared, while immune regulation pathways were reduced, highlighting disrupted innate immune responses. Astrocytes showed mitochondrial and vesicle handling dysfunction, with over-activated antigen presentation via major histocompatibility class II complexes. Both oligodendrocytes and OPCs exhibited transcriptome dysregulations in immune regulation and cytokine signaling, aligning with the neuroinflammatory process in PD. Particularly, OPCs shared protein refolding dysregulation similar to microglia, while oligodendrocytes (not shown in Fig. 1b) displayed an enrichment of dopaminergic neuron differentiation pathways. Suggesting “pan-glial” activation as a central pathological mechanism in PD.

The electrophysiological recordings showed that in comparison to healthy controls the patients with biopsies had a robust increase in beta and decreased narrow gamma power, mirroring the established findings on larger PD cohorts (Fig. 1c) and a marginal significance for increased phase-amplitude coupling. We then evaluated whether there are particular molecular signatures of this abnormal oscillatory activity through a multifactorial association analysis (Fig. 1c; see “Methods” section). Here, distinct modules of genes for each cell type were found to correlate with pathological EEG beta power, gamma power, and their phase-amplitude coupling. An over-representation analysis attested the biological meaning of the modules. Here, the main significant electrophysiological features highly related to alteration in metabolic pathways in microglia and OPCs, including protein folding and refolding, and heat-shock protein response; in accordance to the PD transcriptomic profile above another set of pathways involved ‘adaptive immune response’. Among the immuno-metabolic pathways, the top upregulated genes were: HSP90AA1, HSPA1A, HSPD1, and DNAJA4 (Fig. 1d). The correlations observed with phase-amplitude coupling depicted inflammasome pathways such as ‘lymphocyte-mediated immunity’ and ‘adaptive immune response’, with upregulated genes including: P2RX7 and PRKCB (Fig. 1d); again highlighting “pan-glial” activation.

Our results confirm recent studies suggesting that neurodegeneration is tightly related to impaired metabolism and altered cerebrovascular mechanisms, which fluctuate accordingly to the rapidly adapting microglia.^2^ Our work complements this by adding the implication of glial mitochondrial dysfunction, immune responses, and protein folding, emphasizing the critical role of both neuronal and glial dysfunction in the development and progression of neurological disorders.

Finally, using gene-drug interaction analysis with enrichR, we identified five potential drugs that target the expression of these genes, with NVP-BEP800 showing the highest possible affinity to the upregulated genes (Fig. 1d). NVP-BEP800 is a heat-shock protein 90 (Hsp90) inhibitor that by binding to the ATP-binding site modulates HSP90 client proteins and downstream signaling pathways. HSPs are key in regulating proteoasis, immunological pathways, and regulated cell death. In Parkinsonian mouse models brain tissue injected with pre-formed human wild-type α-synuclein fibrils (PFFs), HSP90 co-localizes with filamentous S129 phosphorylated α-synuclein in ubiquitin-positive inclusions,^3^ while in human brain samples chaperone-encoding transcripts in the substantia nigra, medial temporal gyrus, and amygdala overexpression of HSPA1A and HSP90AA1 and associated with alpha-synuclein,^3^ and inclusion of HSP90β results in transient membrane binding and triggers a remarkable re-localization of α-synuclein to the mitochondria and concomitant formation of aggregates.^4^ Therefore, regulation of HSP chaperones may have a central role in controlling alpha-synuclein function and aggregation as a novel therapeutic approach for PD.

Recent work in animal models of PARKIN deficiency has proposed that accelerating PARKIN‐induced inflammasome degradation by administration of a HSP90 inhibitor reduces the inflammatory response and alleviates neurodegeneration.^5^ In our dataset we have identified further PD-specific dysregulated pathways linked to Hsp90 that could be potentially tested in mice through Hsp90 inhibitors: ‘response to unfolded protein’, ‘response to topologically incorrect protein’, ‘protein folding’, ‘protein refolding’, ‘chaperone-mediated protein folding’.

Overall, our unique ex-vivo dataset offers the first genomic mapping in the single-cell level for the PD cortex, allowing us to associate molecular signals with clinically relevant disease hallmarks. Our findings establish a molecular basis for pan-glial immunometabolism dysregulation as a central mechanism underlying the electropathophysiological activity in PD. Within these cell-specific enriched metabolic pathways and depleted immune pathways key genes are at the interface of the inflammasome and are good candidates for biomarkers and open a new possibility for immunotherapeutic options.

## Supplementary information

Supplementary information

## Data Availability

All data supporting the findings of this study are available in the main text and its supplementary information. Raw data and further information are available from the corresponding authors on request. All analyses were performed using openly available software and toolboxes. For the single-cell RNA data, available R packages (Seurat, DESeq2, WGCNA, GSEA, PANTHER, enrichR) were used. For the EEG data Fieldtrip (https://www.fieldtriptoolbox.org/) and the PAC toolbox (https://github.com/sccn/PACTools) were used.
