# Supplementary material for: Targeting pathological brain activity-related to neuroinflammation through scRNA-seq for new personalized therapies in Parkinson’s disease
Source: Signal Transduct Target Ther. 2025 Jan 10;10:10. doi: 10.1038/s41392-024-02086-7 (PMC11718261; doi:10.1038/s41392-024-02086-7)
Supplement: Supplementary file 1 — Supplementary information [file 41392_2024_2086_MOESM1_ESM.docx]

**Supplementary Materials for**

**Targeting pathological brain activity-related to neuroinflammation through scRNA-seq for new personalised therapies in Parkinson's disease**

**Materials and Methods**

**Human brain tissue**

Before inclusion, all subjects gave their informed to participate in the study. The study was conducted in accordance with the Declaration of Helsinki, and the protocol was approved by the local Ethics Committee.

Fresh human dorsolateral prefrontal cortex (DLPFC) samples were obtained from 14 subjects (mean age: 57.79±15.57 years, 9 females): All participants provided informed consent. Patients included 9 PD (mean age 57.77±15.3 years, mean disease duration = 10.1±5.3, median Hoehn and Yahr 3±1, median UPDRS-III: OFF = 30±8.5, ON = 14±7.6, sample weight range = 50-100 mg) and 5 non-PD (mean age 57.8±16.2, mean disease duration = 13.4±5.6, sample weight range = 50-100 mg; 2 multiple sclerosis, 2 obsessive-compulsive disorder, 1 essential tremor and one dystonia).

Patients underwent clinical examination by a movement disorders specialist (SG) in the Department of Neurology of the University Medical Center of the Johannes Gutenberg University Mainz. Further, patients were tested by a clinical neuropsychologist to exclude cognitive impairment. DLPFC samples were extracted from under the skull borehole before DBS electrodes implantation. No compensation was provided to any patients.

**Sample Processing**

DLPFC samples were washed in Hanks Balanced Salt Solution (HBSS) three times, then stored in GEXSCOPE tissue preservation solution (Singleron Biotechnologies) immediately and transported at 4°C to the laboratory. Afterwards, the sample was processed fresh within 24 h following the manufacturer’s protocols (Singleron Biotechnologies). The microwell SCOPE-chip methodology for high-throughput single-cell sequencing (Singleron Biotechnologies) is less sensible for human neurons, due to cell membrane lesioning during the mechanical and chemical digestion, and to their bigger size compared to other cell types. The same amount of cells was used for each patient. Libraries were sequenced and stored as fastq files.

**Sequencing Data Processing and Downstream Analysis**

CeleScope (version 1.8.1 www.github.com/singleronRD/CeleScope; Singleron Biotechnologies GmbH) was used to process raw reads for single-cell 3′ gene expression using the human genome GRCh38 as reference. The R package Seurat (version 4.3.0) (Satija et al., 2015) was used for quality control (QC) and downstream analysis. WC was performed for each data set individually and included filtering cells based on the number of detected genes and unique molecular identifiers (UMIs) per cell. We also filtered out cells with > 10 % mitochondrial counts.

The Seurat integration workflow (Stuart et al., 2019) was used to harmonise batch effects and facilitate cross-sample comparisons. To integrate the 14 datasets, after prior normalisation and scaling, the ‘FindIntegrationAnchors’ function was used to identify a set of anchors between all Seurat objects in the list, and these anchors were then used to integrate all objects together with ‘IntegrateData’. Finally, the standard workflow for processing scRNA-seq data was applied, including ‘RunPCA’, ‘FindNeighbors’, and ‘FindClusters’, ‘RunTSNE’. The clustering was performed using 50 dimensions and a resolution of 0.5. Assignation of biological identities to the identified cell clusters, was performed manually based on known marker genes from the literature research and a public database (Franzén et al., 2019).

Group (PD vs. non-PD) differential gene expression analysis was conducted on pseudo-bulk samples per cell type using the R/Bioconductor package DESeq2 (version 1.34.0) normalising the counts by library size in the integrated dataset (Love et al., 2014). Functional profiling was performed using GSEA of GO terms related to biological processes (BP) as implemented in the R/Bioconductor package ‘clusterProfiler’ (version 4.2.2) (Wu et al., 2021). Visualisation of the network enrichment analysis used R/Bioconductor package enrichplot (version 1.14.2) (Wu et al., 2021).

Weighted gene correlation networks (WGCNA) to EEG parameters were computed for each cell type using a dedicated R package (version 1.72-1) (Langfelder et al., 2008), based on the differential gene expression analysis output. An unsigned network (individually selected soft power) was calculated, and then used to perform hierarchical. Modules were created with the ‘mergeCutHeight’ function using a cutHeight of 0.4 and the ‘minModuleSize’ equalling 100. Functional profiling of the relevant WGCNA modules was performed using over-representation analysis on GO terms (domain: BP, MF) using PANTHER (Thomas et al., 2022) and visualised using enrichplot (Wu et al., 2021). Then, the ggvenn package (version 0.1.10) was used to highlight the overlapping terms between the two previous analyses (group differences with GSEA and gene-EEG associations with WGCNA) and were visualised using Ridgeplot (ggplot2, version 3.4.3).

We used enrichR (version 3.2)(Kuleshov et al., 2016) to perform gene-drug interaction analysis based on the proteome-wide atlas of drug mechanism of action gene set library (Mitchell et al., 2023) and six identified genes from the last previous step: HSP90AA1, HSPA1A, HSPD1, DNAJA4, P2RX7, PRKCB. P-adjusted-values were used.

**EEG Data Acquisition and Processing**

A total of 91 PD (mean age ± standard deviation: 61.70 ± 11.51 years, 19 females) and 38 non-PD healthy controls (mean age: 61.84 ± 9.53 years, 19 females) were included. EEG signals were recoded using a high-density 256-channel HydroCel Geodesic Sensor Net EEG recording system (EGI Netstation, Eugene) with Cz as reference at a sampling rate of 1000 Hz. The EEG recordings were conducted during a five-minute resting state period with participants seated comfortably in a slightly reclined position, both forearms supported by sturdy armrests, eyes closed.

EEG data processing was performed in MATLAB (version R2019b, Mathworks) using FieldTrip toolbox (version 20220310) (Oostenveld et al., 2011). All channels above the line connecting nasion and Oz were included. Pre-processing included, re-referenciation to the common grand average reference with ‘ft_preprocessing’ and resampling to 250 Hz with ‘ft_resampledata’. Data were then segmented into 4 second long epochs with 50 % overlap, detrended, and filtered (highpass: 1 Hz, lowpass: 95 Hz, bandstop: 47-53 Hz). Data was re-segmented to 1 s non-overlapping windows to remove noisy segments and channels using ‘ft_rejectvisual’. Independent component analysis (ICA) was used to remove muscle activity, eye blinks and eye movements. Initially rejected channels were then interpolated using a weighted average of their neighbours using ‘ft_channelrepair’. After preprocessing, the multitaper frequency transformation using ‘ft_freqanalysis’ with discrete prolate spheroidal sequences and a frequency-smoothing of 7 Hz for frequencies ranging from 1 to 100 Hz in 1 Hz steps across the 1 s long segments was applied to analyse the spectral features of the data. Average beta band power for each participant was calculated as the mean power of frequencies between 13 and 35 Hz and 89 channels of interest covering the fronto-central region. PD patients showed increased beta power (non-PD 0.15±0.11; PD 0.22±0.15; T=2.77, p=0.003; PDb 0.28±0.12, T=2.68, p=0.005). Narrowband gamma power for each participant was first normalised by the average power across channels between 35 Hz and 100 Hz and then calculated as the mean power of frequencies between 60 and 80 Hz and the channels of interest. PD patients showed reduced narrow gamma power (non-PD 0.77±0.19; PD 0.7±0.21; T=1.8, p=0.037; PDb 0.62±0.15; T=2.34, p=0.012).

Cross-frequency coupling was computed as the beta-gamma phase amplitude coupling (PAC), calculated as the modulation index (Canolty et al., 2006) with the “Matlab toolbox for estimating Phase Amplitude Coupling” (find_pac_shf_fdr: frequency for beta phase and frequency for gamma amplitude, https://data.mrc.ox.ac.uk/data-set/matlab-toolbox-estimating-phase-amplitude-coupling). The modulation index was then averaged within each subject across the fronto-central channels and frequencies of interest (beta: 13-35 Hz; narrow gamma band: 60-80 Hz). PD patients showed an increased PAC (0.06±0.03; T=1.81, p=0.036) in comparison to non-PD (0.05±0.03), but marginal in PDb (0.05±0.02; T=1, p=0.05).

**Statistical Analysis**

Given the unique nature of the data, no statistical methods were used to predetermine sample sizes. Statistical analyses for the EEG data were performed using FieldTrip. Group differences in beta power, narrow gamma power, and beta-gamma PAC between PD, PDb and HC were tested using single sided T-tests due to the a priori hypotheses (i.e. increased beta power and beta-gamma-PAC and decreased narrowband gamma power) (Brown et al., 2001). For the scRNA-seq data the statistical analysis was performed using RStudio (version 1.4.1717). WGCNA modules to EEG associations were performed using Pearson correlation. P-values < .05 were considered as significant. For the gene expression level, Wilcoxon test within each cell-type was used to test for differences between PD and non-PD subjects. Bonferroni correction within each cell-type (p-value < .05).

**References:**

Brown, P., et al. Dopamine dependency of oscillations between subthalamic nucleus and pallidum in Parkinson's disease. *J. Neurosci.* **21**, 1033-1038 (2001).

Canolty, R. T., et al. High gamma power is phase-locked to theta oscillations in human neocortex. *Science*, **313**, 1626-1628 (2006).

Franzén, O., Gan, L. M., & Björkegren, J. L. M. PanglaoDB: a web server for exploration of mouse and human single-cell RNA sequencing data. Database (Oxford) (2019).

Kuleshov, M. V., et al. Enrichr: a comprehensive gene set enrichment analysis web server 2016 update. Nucleic acids research, **44**, W90-W97 (2016).

Langfelder, P., & Horvath, S. WGCNA: an R package for weighted correlation network analysis. *BMC Bioinformatics*, **9**, 559 (2008).

Love, M. I., Huber, W., & Anders, S.Moderated estimation of fold change and dispersion for RNA-seq data with DESeq2. *Genome biology* **15**, 1-21 (2014).

Mitchell, D. C., et al. A proteome-wide atlas of drug mechanism of action. *Nat Biotechnol*, **41**, 845-857 (2023).

Oostenveld, R., et al. FieldTrip: open source software for advanced analysis of MEG, EEG, and invasive electrophysiological data. Computational intelligence and neuroscience, 2011, 1-9 (2011).

Satija, R., et al. Spatial reconstruction of single-cell gene expression data. *Nat Biotechnol*, **33**, 495-502 (2015).

Stuart, T., et al. Comprehensive Integration of Single-Cell Data. *Cell*, **177**, 1888-1902.e1821 (2019).

Thomas, P. D., et al. PANTHER: Making genome-scale phylogenetics accessible to all. *Protein Sci,* **31**, 8-22 (2022).

Wu, T., et al. clusterProfiler 4.0: A universal enrichment tool for interpreting omics data. *The innovation*, **2** (2021).
